# The Effects of Negative Pressure by External Tissue Expansion Device on Epithelial Cell Proliferation, Neo-Vascularization and Hair Growth in a Porcine Model

**DOI:** 10.1371/journal.pone.0154328

**Published:** 2016-04-29

**Authors:** Hui-Yi Hsiao, Jia-Wei Liu, Eric M. Brey, Ming-Huei Cheng

**Affiliations:** 1 Department of Plastic and Reconstructive Surgery, Chang Gung Memorial Hospital, Taoyuan, Taiwan; 2 Center for Tissue Engineering, Chang Gung Memorial Hospital, Taoyuan, Taiwan; 3 Department of Biomedical Engineering, Illinois Institute of Technology, Chicago, Illinois, United States of America; 4 Research Service, Hines Veterans Administration Hospital, Hines, Illinois, United States of America; University of New Mexico HSC, UNITED STATES

## Abstract

While pre-treating a fat transplant recipient site with negative pressure has shown promise for increasing the fat survival rate, the underlying mechanisms have not been investigated, partly due to challenges related to immobilization of vacuum domes on large animal subjects. The aim of this study was to examine the effect of negative pressure treatment by External Tissue Expansion Device (ETED) on fat grating recipient sites in a porcine model. The ETED was designed to provide negative pressure on the dorsum of swine. Pressure treatment (-70 mmHg) was applied for 1 or 3 hours every other day for 10 and 20 treatments. The treated areas (3.5 cm in diameter) were harvested and examined for histological changes, vessel density, cell proliferation (Ki67) and growth factor expression (FGF-1, VEGF and PDGB-bb). The application of the ETED increased epidermis thickness even after 1-hour treatments repeated 10 times. The results of Ki67 analysis suggested that the increasing thickness was due to cell proliferation in the epidermis. There was a more than two-fold increase in the vessel density, indicating that the ETED promotes vascularization. Unexpectedly, the treatment also increased the number of hair follicles. Negative pressure provided by the ETED increases the thickness of epidermis section of tissue, cell proliferation and vessel density. The porcine model provides a better representation of the effect of the ETED on skin tissue compared to small animal models and provides an environment for studying the mechanisms underlying the clinical benefits of negative pressure treatment.

## Introduction

Fat grafting is a common practice in cosmetic surgeries for the resurfacing of face wrinkles, scars or depressed deformities and in reconstructive surgeries for breast contouring and radiated breast deformities. While fat grafting is undoubtedly a useful and successful technique, the survival rate of fat graft transfers is still unpredictable, ranging from 20 to 90 percent [1–3]. Previous studies have also shown that fat graft transferred in small volumes displayed a better survival rate than larger volumes [4–6]. When fat graft tissue is transferred in a large volume in the absence of a pedicle, the central portion of the injected fat graft tissue could suffer from the lack of nutrients, which can lead to fat necrosis, calcification, lipo-necrotic cysts and abscess [7]. Kato et al. suggested that the grafted fat undergoes dynamic tissue remodeling in the first three months [7]. Fat grafts first undergo degeneration and proceed to a regeneration stage when the new adipocytes have successfully developed from adipose progenitor cells. The failure replacement by newly generated adipocytes leads to necrosis and oil cyst formation. The survival of fat grafts is highly dependent on the local microenvironment at the recipient site. One effective way to minimize necrosis of the region is to prepare the recipient site prior to transplantation.

Mechanical forces play an important role in regulating cell proliferation and remodeling in many tissues [8]. Previous clinical studies have shown that mechanical forces can be used to promote wound healing [9]. Recently, clinical studies have demonstrated the feasibility of applying sub-atmospheric pressure using external tissue expansion devices (BRAVA, LLC., Miami, Florida) at a recipient site to enhance the survival of injected fat [10, 11]. The mechanism underlying this enhancement is unknown, but an increase in cell proliferation and vascular remodeling has been demonstrated in a murine model [12]. However, the anatomy and physiology of the skin of small mammals are not consistent with that of humans. For example, rodents have a densely packed hair follicles and thin epidermis while human skin has less hair follicles and a thicker epidermis [13, 14]. In addition, rodents have the *panniculus carosus* that further differentiates their skin anatomy from human. However, studies have shown that the epidermis to dermis thickness ratio in swine is similar to the ratio in human skin tissue [13, 15]. However, it is difficult to immobilize vacuum cups on large animals, hindering research aimed at investigating the effect of negative pressure on the fat grafting recipient site. Studies using negative pressure in large animals could provide important insights on the mechanisms relevant to clinical success. In this study, we demonstrate the application of an external tissue expansion device (ETED) in a porcine model, providing insight into the mechanisms underlying the contribution of negative pressure pretreatment of recipient sites to the increase in the survival rate of fat grafts.

## Materials and Methods

### External Tissue Expansion Device (ETED)

**Fig 1:**
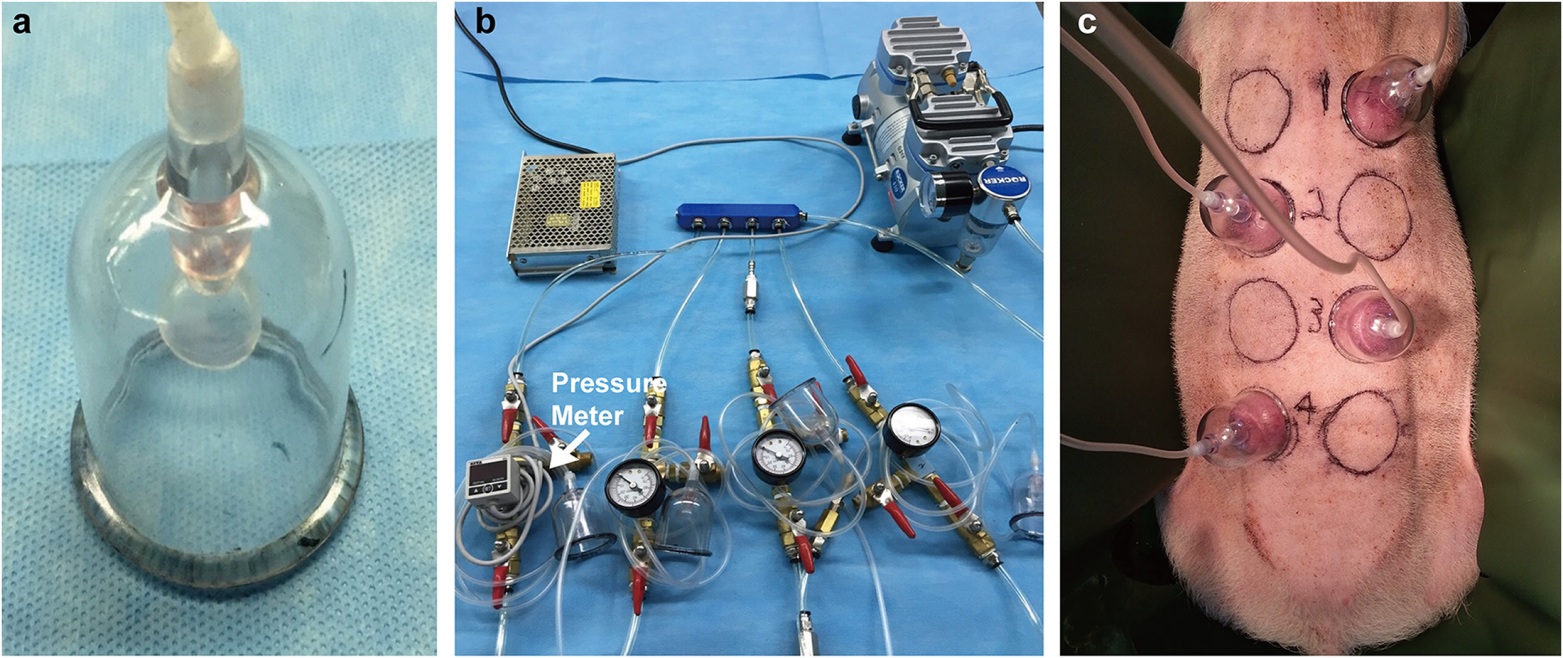
An External Tissue Expansion Device (ETED). It contains (a) a suction cup 3.5 cm in diameter and 5 cm in height that connected to (b) the pump system. The pump system contains the pump that supplies negative pressure for 4 branches that were linked to 4 suction cups. The pressure meter is used to monitor the pressure. (c) The application of the ETED on porcine model.

To generate continuous and uniformly distributed negative pressure on the dorsal skin of swine, a clear plastic dome was applied to the dorsum (Fig 1a). The ETED was selected to provide a better fit for the back of the swine. The size of the suction cup is 3.5 cm in diameter and 5 cm in height. The edges of the ETED were fitted with a silicone gel sheet (Smith & Nephew, Memphis, Tennessee) to reduce the risk of pressure sores. A negative pressure of 70 mmHg was applied through a customized device, which was composed of a pump, pressure meter, 4 branches of tubes and 4 suction cups (Fig 1b). The pump provided continuous negative pressure for the time needed for the experimental design, and the pressure meter allowed accurate monitoring of the pressure. The suction cups were applied on the skin tissue on the dorsum of swine (Fig 1c).

### Animal Model

All animal procedures were carried out in strict accordance with the recommendations in the Guide for the Care and Use of Laboratory Animals of the Chang Gung Memorial Hospital Animal research guideline. The protocol was approved by the Committee on the Ethics of Animal Experiments of the Chang Gung Memorial Hospital in Taiwan (Permit Number: 2013010201). All surgery was performed under isoflorance anesthesia, and all efforts were made to minimize suffering. All pigs spent one month in quarantine prior to the experiments. Three skeletally mature domestic swine were chosen for the ETED study. They were a mixture of Landrace and Yorkshire (LY) (Agriculture Technology Research Institute, Hsinchu, Taiwan). LY pigs are a common pork resource in Taiwan. LY pigs (approximately 15–20 weeks of age) weighing 50~55 kg were used in this study. The skin of LY pig is similar to human skin in terms of color, hair follicles, sweat glands, and subcutaneous fat [16].

After fasting for 8 to 12 h, the LY pigs were injected with atropine (atropine sulfate, Tai-yu, Taiwan) intramuscularly, followed by Zoletil and Rompun at a 1:1 mixture (Zoletil, Virbac, France, 0.55 to 0.80 mg/kg) intramuscularly to achieve the initial stage of anesthesia. Subsequently, gas anesthesia with isoflurane 2.5% to 4.5% was applied, followed by 1% to 3% for maintenance of anesthesia after the completion of intubation. The pigs were placed in the prone position, and the ETED was utilized to provide negative pressure on the dorsal skin. A treatment course consisted of 1 or 3 hours of continuous negative pressure at 70 mmHg. The treatment was repeated every other day 10 or 20 times, resulting in a total experimental period of 20 days or 40 days depending on the number of applications (10 and 20 total treatments, respectively). Each pig were carry out four experimental groups which were 1 hour-10 days (1H-10 Group), 1 hour-20 days (1H-20 Group), 3 hours-10 days (3H-10 Group) and 3 hours-20 days (3H-20 Group). Totally three pigs were performed in this study resulting in 3 biological replicates for each experimental groups. Upon the completion of the treatment, the skin was harvested while the animals were under gas anesthesia with isoflurane 2.5%. An area of the skin (3.5cm in diameter) treated with ETED was harvested by dissecting out the skin tissue including epidermis and dermis layers to ensure inclusion of the entire tissue of interest. Right after harvesting, the animals were euthanized with KCL (2mEg/kg) under gas anesthesia. The specimen was cut into two pieces, one part was fixed in 10% neutral-buffered formalin solution and then paraffin embedded and the other part was flash frozen with liquid nitrogen until total protein extraction was performed.

#### Histological analysis and Immunohistochemisty

Tissues were immediately fixed in 10% neutral buffered formalin overnight and embedded in paraffin according to standard procedures. Tissue sections (5 μm) were cut, mounted on slides and stained with hematoxylin and eosin and Masson’s trichrome. For immunohistochemical analysis, the paraffin-embedded sections were de-paraffinized and rehydrated by incubation in a series of xylene and ethanol solutions, and then washed with deionized water. Antigen retrieval was accomplished by incubating slides in 10 mM sodium citrate (pH 6.0) solution at 95°C for 20 minutes on a hot plate. Slides were left to cool for 20 minutes, washed twice in TBS-Tween (0.025% Triton X-100) and blocked in 10% normal serum with 1% BSA in TBS for 2 hours at room temperature. Primary antibodies, CD31 (1:50 in TBS with 1% BSA), Ki67 (1:200) and keratin-16 (1:50)(Abcam, Cambridge, MA), were applied to the slides and incubated at 4°C overnight. The slides were washed twice in TBS-Tween. Secondary antibodies were applied and incubated at room temperature for 1 h.

### Evaluation

All of the stained sections were digitally imaged using an Axio Scope A1 microscope (Carl, Zeiss, Germany) for analysis. The epidermis thickness and hair follicles in each view area (5 x objective, 1.4 mm x 1 mm area) were measured and counted, respectively. For each tissue section, five view areas were randomly selected and quantified. Cell proliferation was evaluated by calculating the percentage of basal keratinocytes that were Ki67 positive. The blood vessel density was analyzed by counting the number of CD31-positive vessels and dividing the number by the total tissue area.

### Enzyme-linked immunosorbent assay

Proteins were extracted from the tissue samples by incubation in Radio-Immunoprecipitation Assay (RIPA) buffer for at least one hour on ice and centrifuging at 14,000 rpm for 30 minutes at 4°C. The amount of total protein in the supernatant was determined by Bicinchoninic Acid (BCA) assay (Thermo, Waltham, MA). The amounts of vascular endothelial growth factor A (VEGF-A; R&D, Minneapolis, MN, USA), fibroblast growth factor 1 (FGF1; R&D, Minneapolis, MN, USA) and platelet-derived growth factor BB (PDGF-BB; R&D, Minneapolis, MN, USA) were determined by ELISA according to the manufacturer’s instructions.

### Statistical Analysis

The measurements of epidermis thickness, cell proliferation, blood vessel density and hair follicles were evaluated by analysis of variance (ANOVA) with Turkey’s post hoc test (S1 file). Data were expressed as the means ± standard error. A *p* value less than 0.05 was considered statistically significant.

## Results

### Histological analysis

The effect of the ETED was evaluated by applying negative pressure to a porcine model several times. To immobilize the suction cup, pigs required full body anesthesia during the ETED treatment. Four different experimental conditions with various suction periods and frequency were designed. The suction pressure applied when using BRAVA on human skin is approximately 15–33 mmHg. This pressure setting differs from the treatment for wound healing, which usually requires a high negative pressure [17, 18]. The suction pressure originally used for the swine skin was 30 mm Hg, however, the suction cup with negative pressure < 60 mmHg did not stay on the animals (data not shown). A negative pressure of approximately 70 mmHg was determined to be the optimal pressure which kept the suction cup on the back of the skin without causing sudden damage to the skin.

**Fig 2:**
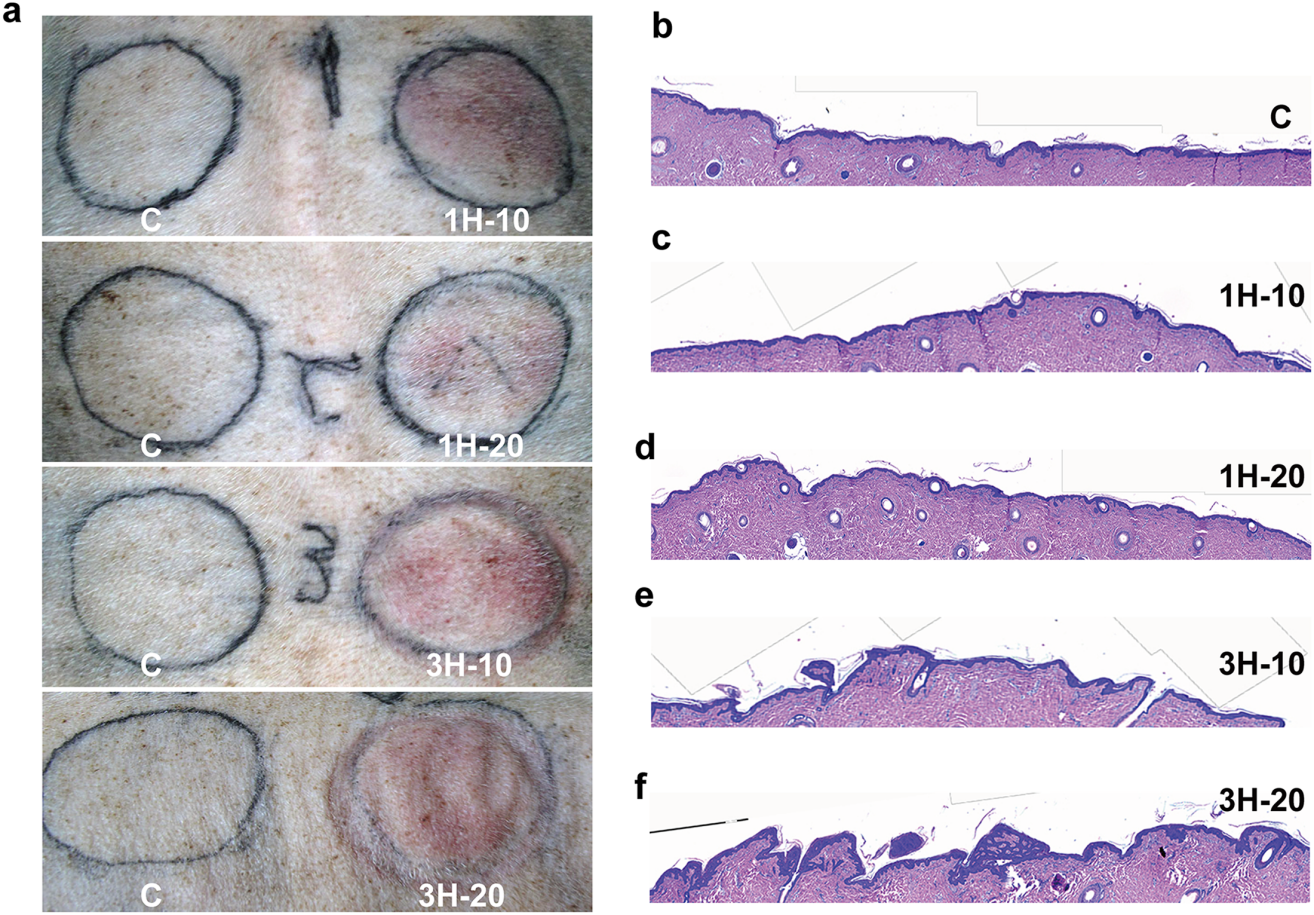
The effects of the ETED on skin tissue. (a) Wrinkle-like skin was observed after ETED treatment, particularly in the 3H-20 group. H&E stains of skin tissue on (b) control group, (c)1H-10 group, (d)1H-20 group, (e) 3H-10 group and (f) 3H-20 group demonstrating that longer ETED treatment results in a more severe effect on the epidermis morphology. C: Control. H: Hour.

One of the goals of skin pretreatment with negative pressure is to create an interstitial space for large volume fat grafting. With the application of the ETED device, the surface of the skin became loose and resulted in wrinkles on the surface of the skin compared to the control group. This phenotype is similar to massage cupping, which has been shown to loosen adhesions and lift connective tissues [19]. The severity of skin loosening seemed to increase with longer application of the ETED (Fig 2a). In H&E histological analysis, the skin tissue treated with the ETED appeared to show more wrinkle folds in the epidermal layer. The 3-hour group (3H-10 and 3H-20 group) showed significant changes in the epidermis compared to their 1-hour counterparts (Fig 2b–2f). Thus, the wrinkle fold increased with longer ETED treatment.

### The thickness and cell proliferation of the subcutaneous layer

**Fig 3:**
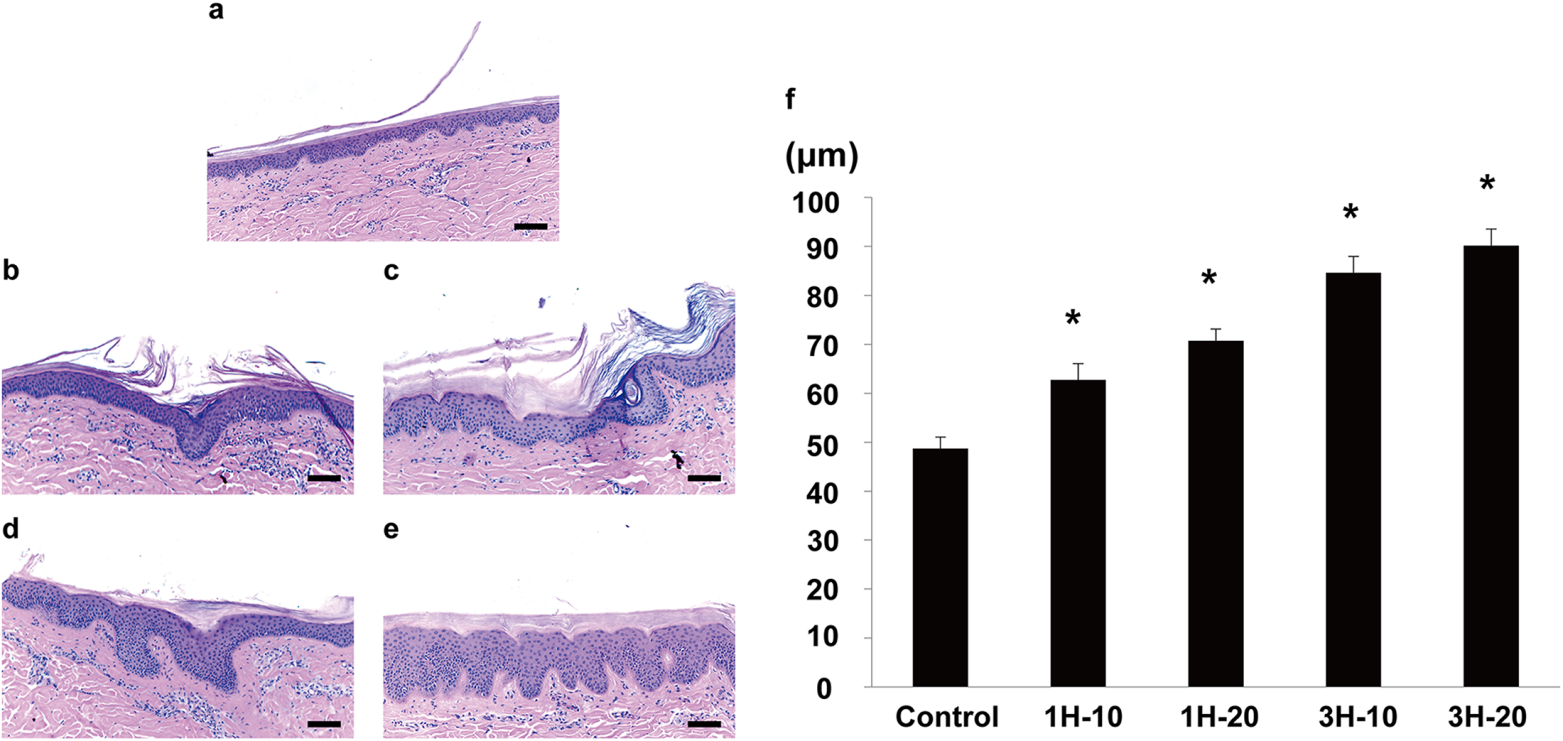
Images of H&E staining on the epidermis and dermis. Samples are from (a) control group, (b) 1H-10 group, (c) 1H-20 group, (d) 3H-10 group and (e) 3H-20 group (Scale bar equals to 100 μm). (f) Quantification of subcutaneous thickness under ETED treatment showing increasing thickness with treatment time. * Indicates statistical significance *p* <0.05.

The skin tissue treated with the ETED was evaluated with H&E to investigate the effect of the ETED on the thickness of the epidermis. The thickness increased with negative pressure (Fig 3a–3e). The thickness of the epidermal tissue in 1H-10 group was significantly larger compared to that of the control group (48.7±2.31 μm vs 62.7±3.39 μm, *p*<0.001). With the extension of the ETED treatment time, the effect on epidermis thickening was more prominent (1H-20 group vs. control group, 3H-10 group vs. control group, 3H-20 group vs. control group; *p*<0.0001 for all comparisons) (Fig 3f). Both 3-hour groups exhibited a significantly thicker epidermis layer compared to their 1-hour counterparts (*p* < 0.00001 for both comparisons). In both 1-hour treatment groups, 10 days of application showed no significant difference compared to 20 days of application. A similar trend was also observed in the 3-hour treatment groups.

**Fig 4:**
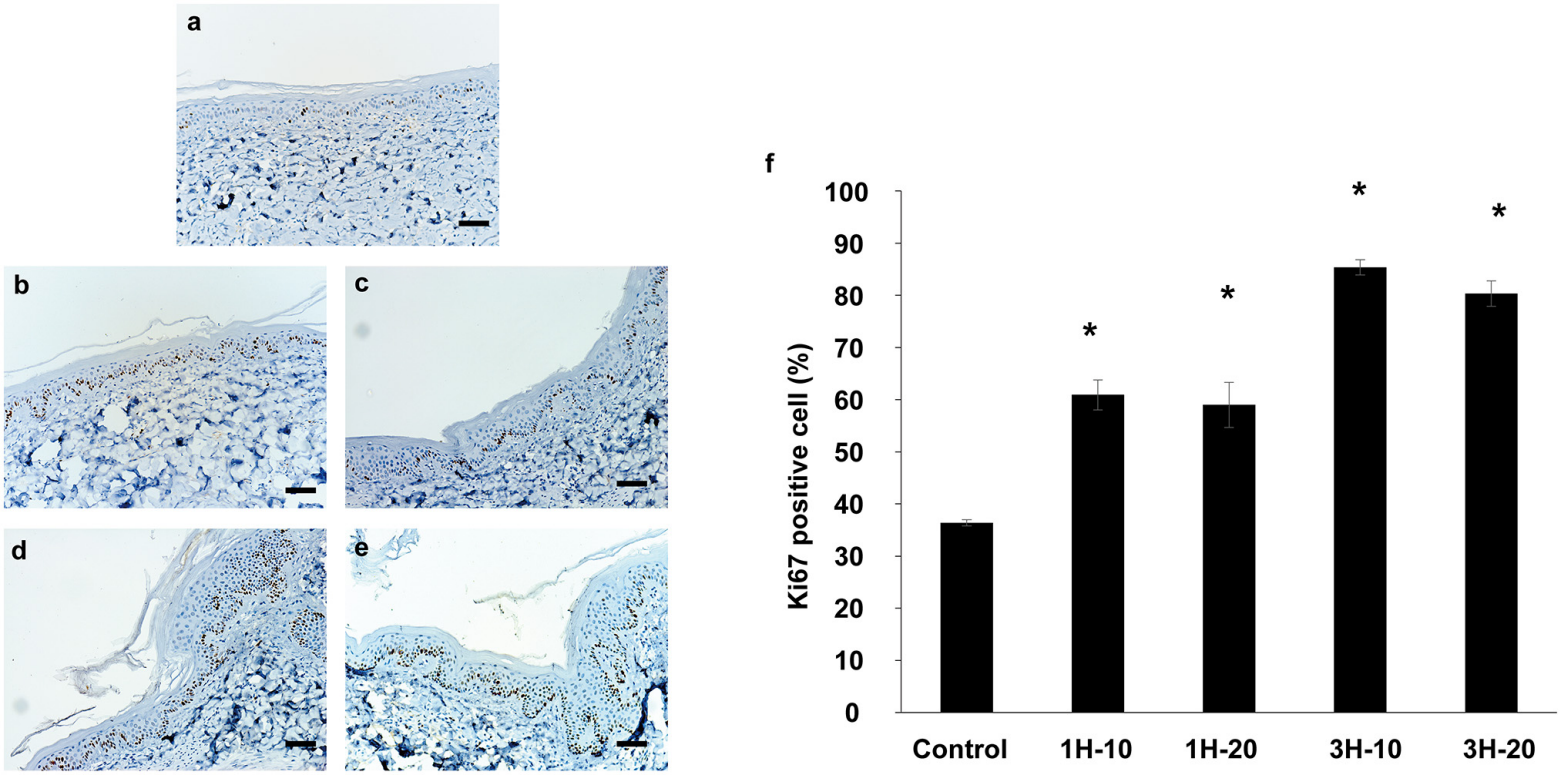
Sections of specimens were stained with Ki67 (brown) to evaluate cell proliferation. Those specimens are from control group (a), 1H-10 group (b), 1H-20 group (c), 3H-10 group (d) and 3H-20 group (magnification, 50x, Scale bar equals to 100 μm). (e) The percentage of Ki67-positive cells. * Indicates statistical significance *p* <0.05.

Cell proliferation was analyzed to determine whether the increasing epidermis thickness results from increased cell proliferation in the epidermis. Ki67 staining, an indicator of cell proliferation, was quantified in the epidermis. The percentage of Ki67-positive cells was higher with ETED treatment compared to the control group (Fig 4a–4e). The 1-hour treatment groups had a higher number of Ki67-stained cells compared to the control group (60.9 ±2.86% and 59.0±4.33% vs. 36.4±0.59%, *p*< 0.01), and the 3-hour groups had a higher number Ki67-positive cells compared to the 1-hour groups (1H-10 vs. 3H-10, *p*< 0.01; 1H-20 vs. 3H-20, *p*< 0.01). Quantitative analysis indicated that the increased percentage of Ki67-positive cells corresponds to a longer ETED treatment (Fig 4f). The results indicated that the increasing epidermis thickness likely results from increased cell proliferation promoted by ETED treatments.

### The effect of the ETED on neo-vascularization

**Fig 5:**
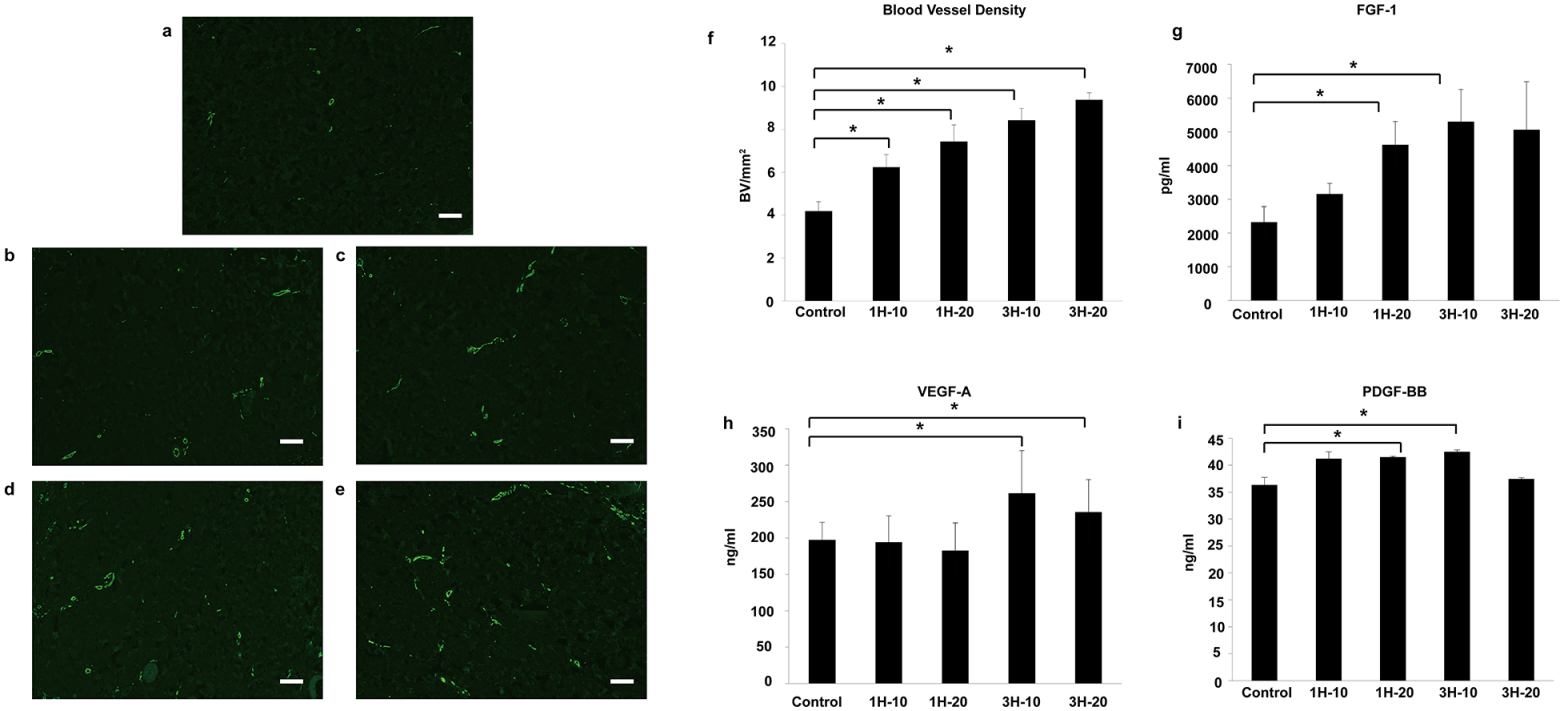
The blood vessels were evaluated by staining for CD 31 (Green). Results are shown for the (a) control group, (b) 1H-10 group, (c) 1H-20 group, (d) 3H-10 group and (e) 3H-20 group (Scale bar equals to 100 μm). Animals treated with the ETED showed an increase in blood vessel density (f) and higher levels of FGF (g), VEGF-A (h) and PDGF-BB (i) compared to the control group. * Indicates statistical significance *p* <0.05.

Tissue sections were also stained for CD31 to evaluate the influence of the ETED on blood vessel density (Fig 5a–5e). The histological data indicated that the 1-hour ETED-treated group resulted in a significantly higher number of blood vessels compared to the control group (6.2±0.59 BV/mm^2^, 7.4±0.78 BV/mm^2^ vs 4.2±0.44 BV/mm^2^, p<0.001) (Fig 5f). Similar results were also found in the 3-hour treatment groups compared to the control (8.4±0.55 BV/mm^2^, 9.4±0.33 BV/mm^2^ vs 4.2±0.44 BV/mm^2^, p<0.001) (Fig 5f). Even a 1-hour ETED application was sufficient to increase blood vessel density. Next, protein analysis was performed to provide further insight into the signals expressed in response to the ETED. The levels of the angiogenesis factors FGF-1, VEGF-A and PDGF-BB were examined. No significant difference in FGF-1, VEGF-A and PDGF-BB levels was observed in the 1H-10 group (p = 0.2, p = 0.95, p = 0.06, respectively; Fig 5g–5i). However, the extension of the ETED treatment period was sufficient to increase the levels of the angiogenesis factors (Fig 5g–5i). The 3H-10 group exhibited the highest increase in FGF-1, VEGF-A and PDGF-BB levels among all experimental groups (*p* = 0.05, *p* = 0.04, *p* = 0.01, respectively). These results suggest that the increase in vessel density was primarily due to an increase in angiogenesis factor levels promoted by the application of the ETED.

### Quantification of hair follicles

**Fig 6:**
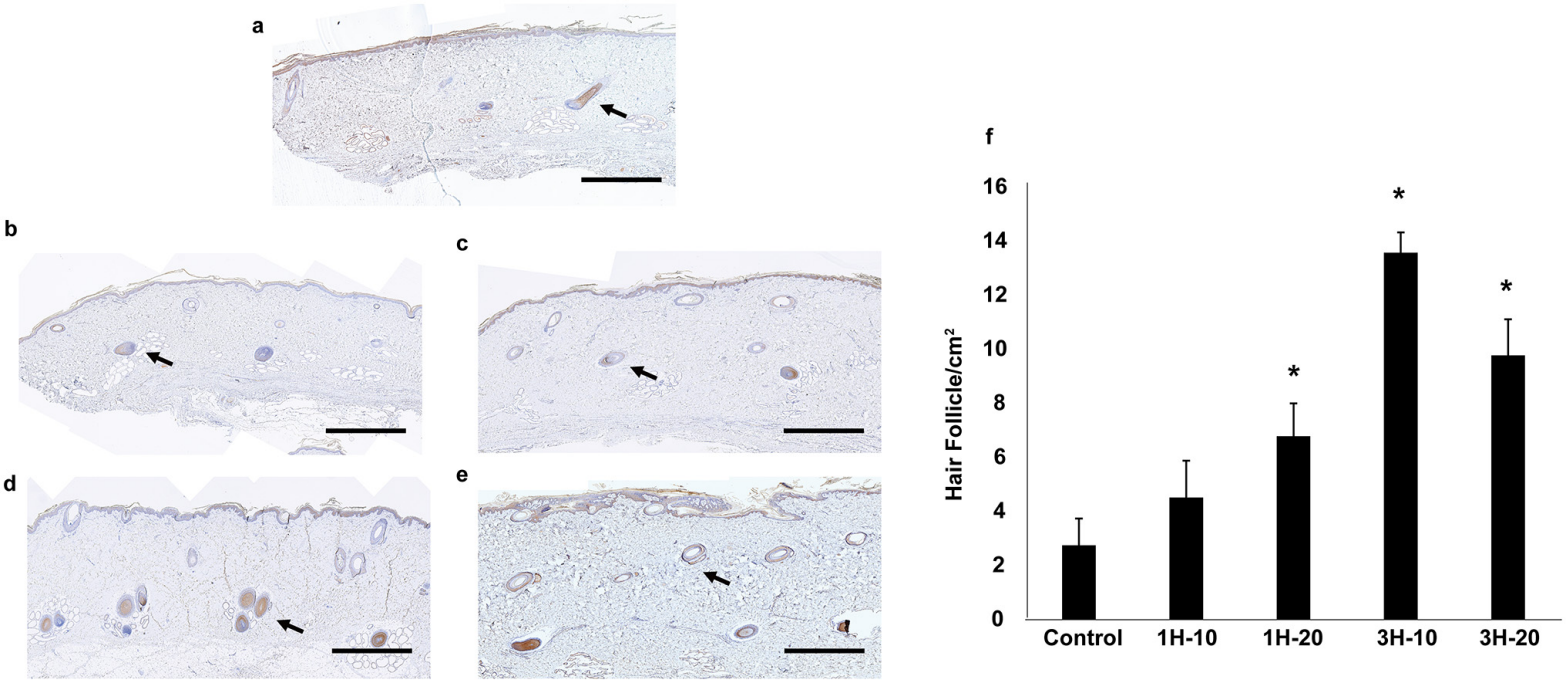
Hair follicle evaluation analysis. Keratin-16 stains (brown) of the specimens from (a) control group, (b)1H-10 group, (c)1H-20 group, (d)3H-10 group and (e) 3H-20 group. The presented images were assembled by approximately 20 images, scale bar is approximately 1 mm. (f) Quantification of hair follicle density. * Indicates statistical significance *p* <0.05. Arrow indicates the location of hair follicles.

Another interesting observation with ETED treatment was the increase in the number of hair follicles. The number of hair follicles stained for keratin-16, a marker of hair follicles, was quantified [20]. An increase in the number of hair follicles was observed in all ETED-treated groups compared to controls except in the 1H-10 group. (1H-20, 3H-10, 3H-20 vs. control; 7.5±1.34 HF/cm^2^, 15±0.83 HF/cm^2^, 10±1.48 HF/cm^2^ vs 3.05±1.09 HF/cm^2^; p<0.05; Fig 6a–6e). Longer ETED treatment resulted in an increase in the number of hair follicles (Fig 6f).

## Discussion

This study investigated the effect of the ETED on skin in a porcine model. The results provide insight into the potential mechanisms underlying the success of the ETED treatment which may be used to improve the clinical outcome of fat grafting. After repeated testing and optimization of the suction conditions and experimental procedures, optimal conditions were identified that allowed us to examine the effect of the ETED in a large animal model. Compared to small animal models, the porcine model is more representative of the human system. With low negative pressure and short periodical treatment, wrinkle folds were observed on the epidermis, resulting in an increase in the interstitial space. These results are consistent with clinical findings and suggest that the space treated with the ETED could provide an environment for large fat grafting.

The thickness of the epidermis increased with treatment duration. Staining for proliferating cells (Ki67-positive) suggested that the thickening of the epidermis was the consequence of a higher cell proliferation rate induced by ETED treatments, rather than the recruitment of adjacent skin tissue. Similar observations on highly proliferative epidermal tissue with negative pressure application were made in a murine model [12].

Increased blood vessel density was also observed with ETED treatment. One-hour ETED treatment was sufficient to elevate the blood vessel density. Increased levels of growth factors involved in the angiogenic cascade, including FGF, VEGF and PDGB-BB, further support a pro-angiogenic state resulting from ETED treatment. The levels of angiogenic factors were higher in the 3-hour ETED treatment group, suggesting that the longer the use of the ETED, the greater the vascular remodeling.

High negative pressures and longer application times had positive effects in our study, but may not represent the best conditions for applications focused on wound healing. Cao et al. reported that 180 mmHg negative pressure was better for wound healing compared to 120 and 240 mmHg treatments [21]. Finding the optimal settings of negative pressure therapy is crucial to obtain the best effects. A specific goal should be identified, however, it is not clear if the goal for fat grafting should be an increase in blood vessel density, volumetric space or another outcome. It is well known that adipose tissue requires a high density of functional blood vessels for survival post transplantation. In this study, the 3-hour treatment groups demonstrated increased epidermis thickness, blood vessel density and expression of angiogenesis factors. However, the 3H-20 group displayed similar effects as the 3H-10 group and lower levels of FGF-1 and PDGF-BB. These results indicate that the 3H-10 group appears to be the optimal condition for pre-treatment of the recipient site for future fat grafting.

Along with an increase in blood vessel density, we observed an increase in the number of hair follicles with ETED treatment. Treatment with negative pressure results in hypoxia, inflammation, ischemia and edema in the skin [22]. There are a number of possible explanations for the increase in the number of hair follicles. First, angiogenesis is associated with active hair growth [23] and VEGF, a key component in angiogenesis, has been reported to act as a mediator of hair follicle growth and cycling [24]. When combined with our findings, the enhancement in angiogenesis in the recipient site may promote hair growth by triggering a change from the hair resting stage to the active stage in the hair cycle. Another possible explanation may be that the negative pressure resulted in hypoxia [22]. Prostaglandin F2 alpha is one of four bioactive prostaglandins that mediates lipid homeostasis and also regulates the inflammatory response [25]. It is abundantly released by the endothelium of hypoxic or ischemic tissues [26]. Moreover, it has been suggested that prostaglandin F2 alpha is active during the early hair growth stage (Anagen) in eyelash growth research [27]. Prostaglandin F2 was also found to have stimulatory effect on hair follicles and follicular melanocytes in a murine model [28]. Those studies suggested that hypoxia or ischemia of the microenvironment may promote hair growth through elevating prostaglandin F2 levels. However, the precise mechanism underlying this observation requires further investigation.

## Conclusion

The main purpose of this study was to develop a porcine model for investigating the effect of the ETED on the recipient site. This model and its results may be used to develop strategies to enhance the survival rate of large volume fat grafting. With more similarities to the human skin, the porcine model allows a much better understanding of the mechanisms of ETED treatment. Our findings suggest the positive impact of ETED treatment on fat graft recipient site may result from increased angiogenesis. Furthermore, the accompanied results of ETED treatment on stimulating the hair follicles may contribute to a potential hair lost treatment.

## Supporting Information

S1 File The evaluation of ETED effect on skin tissue. The evaluation of epidermis thickness, cell proliferation, angiogenesis and hair follicle.
(PDF)
