# Supplementary material for: The Effects of Negative Pressure by External Tissue Expansion Device on Epithelial Cell Proliferation, Neo-Vascularization and Hair Growth in a Porcine Model
Source: PLoS One. 2016 Apr 29;11(4):e0154328. doi: 10.1371/journal.pone.0154328 (PMC4851423; doi:10.1371/journal.pone.0154328)
Supplement: S1 File — The evaluation of epidermis thickness, cell proliferation, angiogenesis and hair follicle. (PDF) [file pone.0154328.s001.pdf]

Epithelial thickness (μ m)

		Control	1h-10	1h-20	3h-10	3h-20
Pig 3						
	1	45.06	46.42	72.93	74.59	86.14
		38.51	37.76	47.16	57.42	92.35
	2	39.26	52.39	48.17	62.61	64.26
		43.49	55.23	65.8	64.4	81.92
	3	47.82	59.47	56.94	71.65	67
		38.75	37.47	63.18	90.11	83.83
	4	37.12	48.37	53.26	74.48	64.73
		40.22	36.64	61.17	72.45	76.86
	5	43.17	61	72.55	67.42	70.23
		41.73	53.86	59.14	61.04	68.07
Pig 4						
	1	56.02	71.72	68.6	87.45	94.42
		35.69	52.34	72.75	91.5	95.78
	2	50.86	66.56	69.81	92.57	97.73
		32.88	54.14	74.05	86.72	113.74
	3	40.78	52.98	92.43	80.75	93.74
		43.56	56.86	68.07	79.8	97.68
	4	37.49	70.71	95.98	88.51	99.1
		47.82	58.47	75.05	91.56	103.44
	5	45.71	69.17	67.71	101.2	92.19
		50.04	63	71.35	100.42	91.52
Pig 5						
	1	58.47	67.51	77.89	92.87	127.05
		57.75	87.33	88.66	84.33	106.96
	2	62.9	94.41	68.33	101.18	96.81
		57.19	74.51	84.22	79.95	86.27
	3	63	72.85	82.51	107.59	98.12
		61.17	73.55	63.94	101.31	89.8
	4	63.83	71.2	69.39	97.68	86.94
		57.63	78.57	84.33	80.18	96.42
	5	67.26	77.37	70.24	95.52	88.44
		56.82	79	77	100.05	92.4

Average

Control	1h-10	1h-20	3h-10	3h-20
41.785	42.09	60.045	66.005	89.245
41.375	53.81	56.985	63.505	73.09
43.285	48.47	60.06	80.88	75.415
38.67	42.505	57.215	73.465	70.795
42.45	57.43	65.845	64.23	69.15
45.855	62.03	70.675	89.475	95.1
41.87	60.35	71.93	89.645	105.735
42.17	54.92	80.25	80.275	95.71
42.655	64.59	85.515	90.035	101.27
47.875	66.085	69.53	100.81	91.855
58.11	77.42	83.275	88.6	117.005
60.045	84.46	76.275	90.565	91.54
62.085	73.2	73.225	104.45	93.96
60.73	74.885	76.86	88.93	91.68
62.04	78.185	73.62	97.785	90.42

3 pig average

	Control	1h-10	1h-20	3h-10	3h-20
Epithelial thickness(μ m)	48.7	62.7	70.8	84.6	90.1
SE	2.31	3.39	2.37	3.34	3.44
P-value		0.002	0.0001	0.0001	0.0001

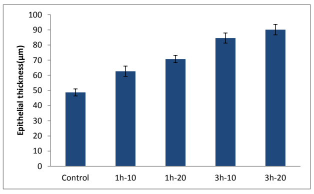

HSD[.05]=11.94; HSD[.01]=14.44

M1 vs M2	P<.05
M1 vs M3	P<.01
M1 vs M4	P<.01
M1 vs M5	P<.01
M2 vs M3	nonsignificant
M2 vs M4	P<.01
M2 vs M5	P<.01
M3 vs M4	P<.05
M3 vs M5	P<.01
M4 vs M5	nonsignificant

PIG#3	contorl	1hr-10times	1hr-20times	3hr-10times	3hr-20times
	31	70	92	107	115.6
in 1.4mm2	22.1	50	65.7	76.4	82.57

PIG#4	contorl	1hr-10times	1hr-20times	3hr-10times	3hr-20times
	37	80.2	88	114.2	98.4
in 1.4mm2	26.4	57.2	62.8	81.5	70.2

pig#3 ki 67 number	46	52	75	120	125
	23	82	105	91	101
	24	76	96	110	121
pig#4	43	45	103	121	99
	33	62	100	120	98
	48	101	99	133	88
	27	111	67	117	100
	34	82	71	80	107
pig#5	85	100	107	119	136
	71	105	104	138	134
	76	97	131	158	110
	51	104	110	148	139
	62	80	92	122	140
	47.9	84.4	96.9	121.3	115.2
1.4 mm2	34.2	60.3	69.2	86.6	82.3
SD	5.7	5.9	4.9	5.8	5.0
P		0.0002	0.0001	0.0001	0.0001

1H-10:1H20	0.1100
3H10:3H20	0.4400
1H10:3H10	0.0002
1H20:3H20	0.0200
1H:3H	0.0001

Ki-67	C	1h-10	1h-20	3h-10	3h-20
	34.2	60.3	69.2	86.6	82.3

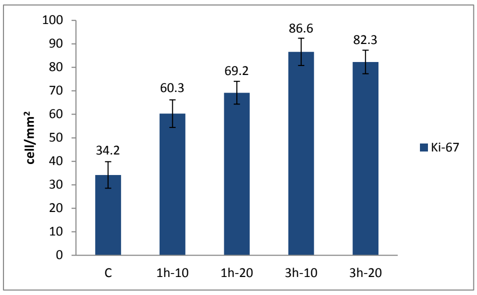

C		1h-10		1h-20		3h-10		3h-20	
ki-67	whole cell	ki-67	whole cell	ki-67	whole cell	ki-67	whole	ki-67	whole cell
67	187	134	261	277	521	273	300	297	362
92	243	123	192	193	260	462	552	198	255
66	185	141	245	214	343	260	310	165	228
92	244	196	302	139	280	287	346	211	255
84	241	145	217	151	271	221	259	219	252

	C	1h-10	1h-20	3h-10	3h-20
	35.8	51.3	53.2	91.0	82.0
	37.9	64.1	74.2	83.7	77.6
	35.7	57.6	62.4	83.9	72.4
	37.7	64.9	49.6	82.9	82.7
	34.9	66.8	55.7	85.3	86.9
AVERAGE	36.4	60.9	59.0	85.4	80.3
	0.59	2.86	4.33	1.46	2.45

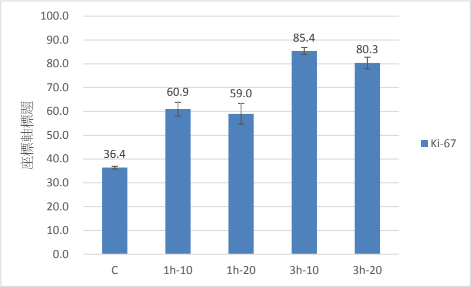

	control	1hr-10 times	1hr-20times	3hr 10 times	3hr-20 times
pig3	2.8	6.7	7.5	8.7	9.2
pig4	3.57	5	7.14	9.14	9.57

	C	1hr-10	1hr-20	3hr 10	3hr-20
PIG#3	4	6	4	10	12
	5	8	15	12	12
	2	10	10	15	13
	3	9	12	12	13
	6	14	12	12	15
PIG#4	6	5	5	6	14
	5	11	6	16	10
	3	9	12	13	17
	4	6	16	13	12
	7	4	11	16	14
PIG#5	11	13	9	9	14
	7	11	14	10	14
	11	8	11	12	12
	7	9	13	11	10
	7	8	6	10	15
MEAN	5.9	8.7	10.4	11.8	13.1
SE	0.44	0.59	0.78	0.55	0.33
	C	1h-10	1h-20	3h-10	3h-20
Density	4.2	6.2	7.4	8.4	9.4

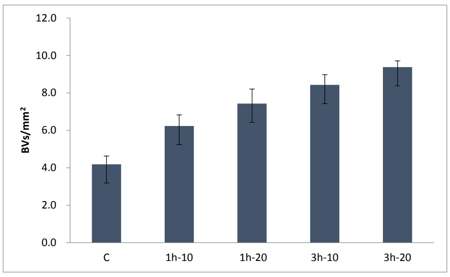

Blood Vessels

	C	1hr-10	1hr-20	3hr 10	3hr-20
P value		0.007	0.0006	0.0001	0.0001

1H-10:1H20	0.17
3H10:3H20	0.16
1H10:3H10	0.05
1H20:3H20	0.05
1H:3H	0.01

PIG	contorl	1hr-10time	1hr-20time	3hr-10time	3hr-20times	
	2	6	6	10	8	
	2	4	7	10	10	
	4	3	6	11	8	
	2	2	4	11	7	
	1	3	4	12	6	
total	11	18	27	54	39	
mean	2.2	3.6	5.4	10.8	7.8	(HF/7.2 MM2)
	3.05	5	7.5	15	10.8	(HF/CM2)
SD	1.09	1.51	1.34	0.83	1.48	

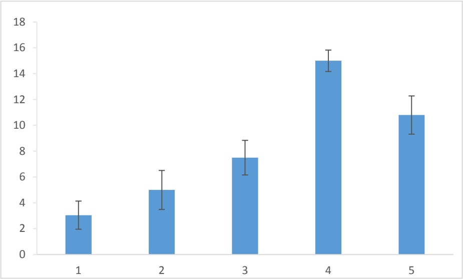

HSD[.05]=2.43; HSD[.01]=3.04

M1 vs M2	nonsignificant
M1 vs M3	P<.01
M1 vs M4	P<.01
M1 vs M5	P<.01
M2 vs M3	nonsignificant
M2 vs M4	P<.01
M2 vs M5	P<.01
M3 vs M4	P<.01
M3 vs M5	nonsignificant
M4 vs M5	P<.05
